# Investigating the regional effect of the chemical shift displacement artefact on the J‐modulated lactate signal at ultra high‐field

**DOI:** 10.1002/nbm.4440

**Published:** 2020-11-02

**Authors:** Carolina C. Fernandes, Bernard Lanz, Chen Chen, Peter G. Morris, Carlos G. Salmon

**Affiliations:** ^1^ Sir Peter Mansfield Imaging Centre University of Nottingham Nottingham Nottinghamshire NG7 2RD United Kingdom; ^2^ Department of Physics University of Sao Paulo Riberao Preto Sao Paulo Brazil

**Keywords:** chemical shift displacement artefact, J‐modulation, lactate, MR spectroscopy, product operator formalism, STEAM

## Abstract

The present work aims to show the applicability of an analytical model for the optimisation of the STEAM sequence timing parameters for lactate detection at ultra high‐field. The effects of the chemical shift displacement artefact on the J‐modulated signal for a weakly‐coupled spin system were considered in the three applied directions of field gradients and the product operator formalism was used to obtain expressions for the signal modulation in each compartment of the excited volume. The validity of this model was demonstrated experimentally at 7 T in a phantom and acquisitions with optimised parameters were performed on a healthy volunteer. The spectra acquired with TE = 144 ms with the optimised mixing time and TE = 288 ms showed easily detectable lactate peaks in the normal human brain. Additionally, the acquisition with the longer TE resulted in a spectrum with less lipid/macromolecular contamination. The simulations shown here demonstrated that the proposed analytical model is suitable for correctly predicting the resulting lactate signal. With the optimised parameters, it was possible to use a simple sequence with sufficient signal‐to‐noise ratio to reliably distinguish lactate from overlapping resonances in a healthy brain.

## Graphical abstract

The applicability of an analytical model for predicting the resulting lactate signal, as well as determining the localisation of the signal throughout the excited region is shown for the STEAM sequence. This three‐dimensional model is particularly useful in the case of an inhomogeneously distributed metabolite and allows for the optimisation of the sequence timing parameters. This work demonstrates that it is possible to use a standard sequence for detecting lactate with sufficient SNR and minimal contamination from lipids and macromolecules, in a healthy brain at ultra high‐field.

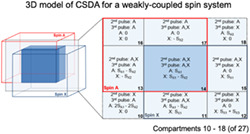

**Abbreviations**
CSDA: chemical shift displacement artefact
MM: macromolecules
SAR: specific absorption rate
TM: mixing time
VOI: volume‐of‐interest

## 1 INTRODUCTION

The measurement of lactate concentration has generated great interest over the years due to its role as a potential indicator for several conditions associated with abnormal anaerobic metabolism. Elevated concentrations of lactate, which can exceed 10 mM, are observed in situations where blood flow, hence also oxygen supply, might be restricted, such as in ischemic stroke^1^ and tumours.^2^ Transient increases in lactate levels have also been observed in the human brain following neuronal activation^3^, ^4^, ^5^ and hypoxia.^6^

Lactate detection with ^1^ *H* MRS using short TE sequences is challenging due to its low concentration in normal brain tissue (∼ 1*mM*) and overlap with signals of higher concentration compounds, namely macromolecules (MM) and lipids. Lactate is a weakly‐coupled spin system due to the spins interactions between the methyl and methine groups. To resolve the lactate methyl signal at 1.31 ppm from its overlapping resonances, some MRS methods exploit J‐modulation effects in combination with the use of a long TE to reduce the intensity of the MM and lipid signals. The PRESS^7^ sequence has been widely used for lactate detection in a clinical setting (field strengths of 3 T and lower). In this spin‐echo based experiment, J‐evolution operates on the methyl group, which is refocused by the 180 ° pulses, to obtain an inverted doublet peak at a TE of 144 ms (1/J, J = 6.933 Hz).

However, ultra high‐field, which offers increased SNR and spectral resolution, imposes power and hence bandwidth restrictions due to specific absorption rate (SAR) limitations. Therefore, the PRESS sequence is associated with a large chemical shift displacement artefact (CSDA), as a result of the limited bandwidth of the slice‐selective refocusing pulses. On the other hand, the STEAM^8^, ^9^ sequence minimises the CSDA effects in comparison to the PRESS sequence, due to the larger bandwidth of the 90° pulses relative to the 180° pulses, which makes the former a popular sequence for use at higher field strengths. To further minimise the CSDA effects, the semi‐LASER^10^ sequence, which uses adiabatic refocusing pulses to address the bandwidth limitation at ultra high‐field, has been employed in several studies for lactate measurement.^5^, ^11^ However, this sequence might only be available on certain scanners as part of research packages.

The modulation in the STEAM sequence arising from scalar coupling is governed by both TE and mixing time (TM). The optimisation of the STEAM sequence timing parameters has been investigated for the improved acquisition of scalar‐coupled metabolites at ultra high‐field, such as glutamate.^12^, ^13^, ^14^ For lactate measurement, previous work reported an analytical expression derived for the STEAM signal modulation in a weakly‐coupled spin system of the lactate form,^15^ *AX*_3_ , where A corresponds to the proton in the methine group and X to those in the methyl group. However, the effects of the CSDA were not included in the latter, it being assumed that both chemical groups experience all 90° pulses.

Although STEAM simulations performed at 4 T with a TE of 70 ms have not shown substantial localisation artefacts for weakly‐coupled spin systems,^16^ the effects of the CSDA should be taken into account when using longer TEs (144 ms) at higher field strengths. Analytical solutions for the response of the STEAM signal have been obtained considering the effect of the CSDA in two dimensions.^17^ Numerical solutions incorporating two^18^ and three‐dimensional^16^ localisation have also been implemented.

However, there is no complete three‐dimensional analytical model with the consideration of the CSDA for assisting the optimisation of the STEAM sequence in the detection of weakly‐coupled spin systems ( *AX*_*n*_ ). The model derived in this work enables the accurate determination of the signal as a function of TE, TM and pulse bandwidth for any *AX*_*n*_ metabolite, including lactate (*n*=3), as well as the localisation of the signal throughout the excited region. This model was validated with a phantom experiment and was also applied to the optimisation of the STEAM sequence timing parameters for lactate detection in the human brain.

## 2 EXPERIMENTAL

### 2.1 Theory

**FIGURE 1:**
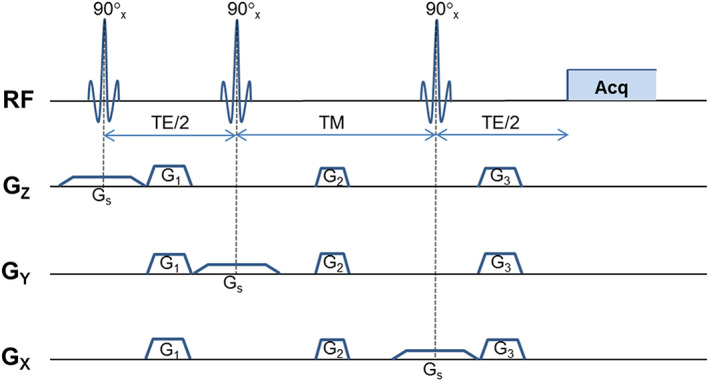
Schematic diagram of a standard STEAM sequence. Three 90° pulses are applied in conjunction to generate a stimulated echo. Each of the RF pulses is applied concurrently with slice selection gradients mutually orthogonal to each other ( *G*_*s*_ ). Dephasing/rephasing gradients *G*_1_ and *G*_3_ are introduced during *T* *E*/2 intervals, while *G*_2_ gradients, which crush unwanted coherences, are added during the TM interval

**FIGURE 2:**
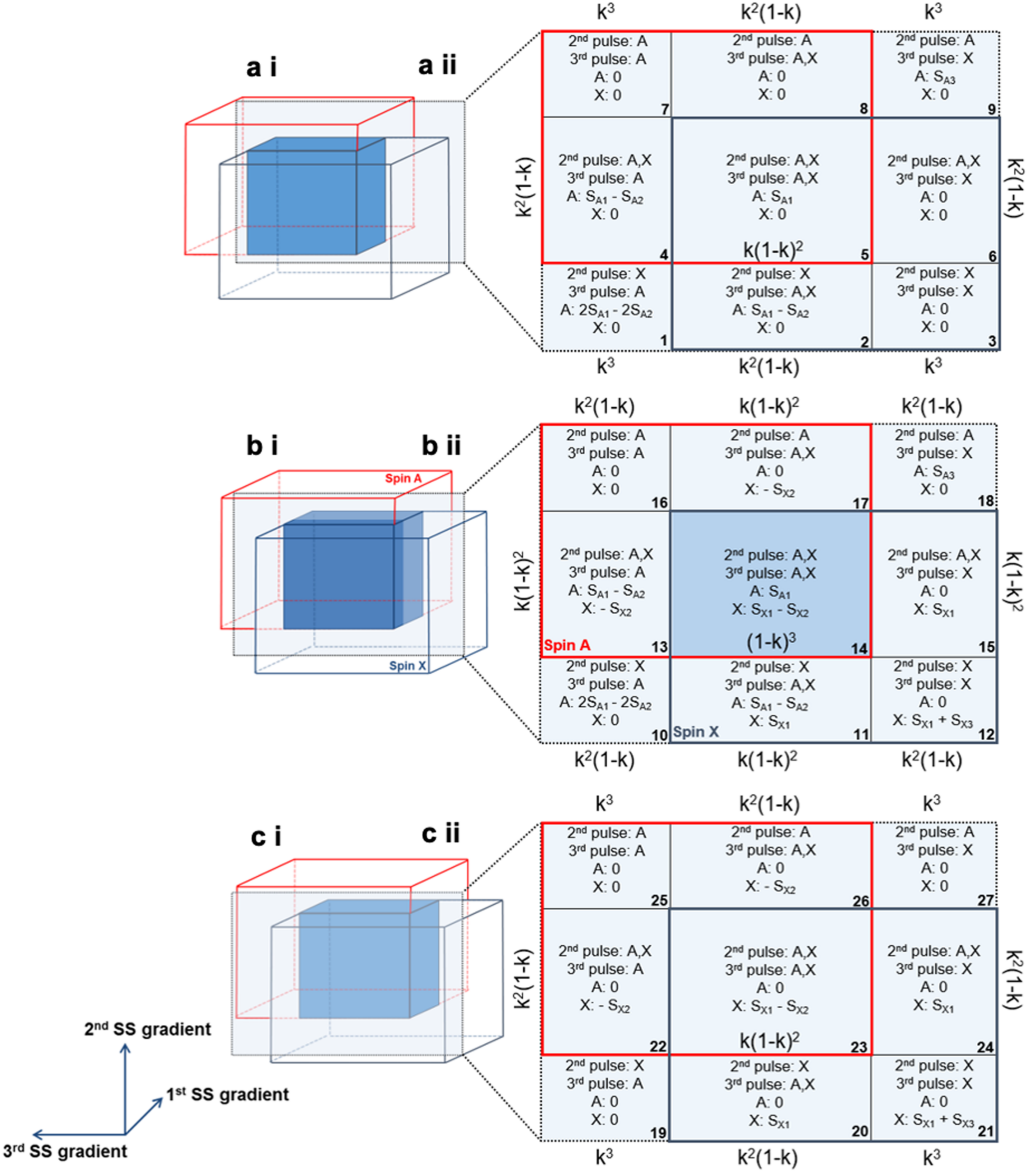
Representation of the CSDA effects in three dimensions on the weakly‐coupled spin system signal during a STEAM sequence. The excited volume can be decomposed into three regions in each slice selection (SS) direction, two where only one of the spins experiences the pulse and one in which both do. The location of the central plane, in which A and X spins are affected by the first pulse, is illustrated in b (i) and the signal from each compartment (labelled 10‐18) is shown in b (ii). The location of the planes in which only the A or X spins experience the first pulse is shown in a (i) and c (i), respectively. The central compartment, shown in darker blue in a‐c (i) and identified by the number 14 in b (ii), is referred to as the ‘ideal condition’ in which both A and X spins experience all three pulses. The signal contributions yielding from each chemical group are shown for each compartment in a‐c (ii) together with the weightings for the calculation of the partial volumes, where *k* is the ratio between the chemical shift difference and RF bandwidth

The STEAM sequence (Figure 1) is composed of three slice‐selective 90° pulses, each of which is subject to a CSDA. The excited volume, defined as the region in which at least one of the A or X spins is excited by at least one of the RF pulses, can be segmented into partial volumes, according to the excitation pattern. For each direction there are three possible patterns of excitation, two in which only one of the spins experiences the pulse (A or X spins) and one in which both spins experience the pulse. Therefore, the excited volume comprises a total of 3x3x3 = 27 compartments (Figure 2).

The product operator formalism was used to derive expressions for the X spin signal from each compartment as a function of TE and TM, following the convention defined in.^19^ Signal modulation in the central compartment (number 14 in Figure 2b (ii) and shown in darker blue in Figure 2b (i) has been previously described in.^15^ It is referred to as the ‘ideal condition’, in which both chemical groups experience all three RF pulses. This work extends the analysis to include the remaining compartments, which is required to fully describe the three‐dimensional model. A similar approach to^15^ was adopted here: all 90° pulses were assumed to be ideal selective, applied with a phase of 0°. This means that their bandwidth profile has a sharp cut‐off, i.e., all spins within the bandwidth experience the same flip angle, whereas those outside the bandwidth are unaffected.

After the application of the first pulse, which transforms longitudinal (L) into transverse magnetisation (single‐quantum coherence, SQ), each of the spins will undergo chemical shift and J‐coupling evolution during TE/2. The objective is to determine the X spin signal: some of this arises directly from excitation of X spins, some is transferred to the X spins from the excited A spins. Thus, A spins terms that do not contribute to the transfer of transverse magnetisation to the X spins or will generate higher‐order coherences in the next interval are excluded. After the second pulse, the transverse magnetisation is converted to longitudinal, single‐ and multiple‐quantum coherences terms. Only those terms insensitive to the gradient *G*_2_ (Figure 1) applied in the TM period will survive, i.e., zero‐quantum coherences (ZQ) and longitudinal magnetisation; transverse and higher‐order coherences are dephased by the gradient. The final pulse is used to convert the longitudinal and zero‐quantum coherences back to single‐quantum terms, which can be detected in the acquisition period. The dephasing created by the gradient *G*_1_ in the first TE/2 interval is recovered by the gradient *G*_3_ present in the last TE/2 interval (Figure 1). In addition, only in‐phase signal at the X spins resonance and detected in the *y*‐axis was considered. The possible coherence pathways contributing to the X spin resonance (top) and the respective signals (bottom) are: 

(1)

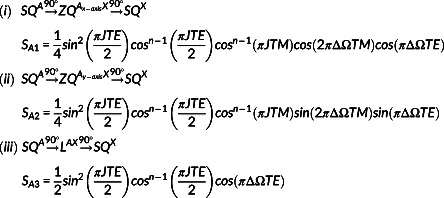

and 

(2)

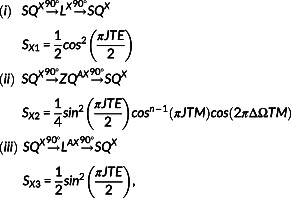

where ΔΩ is the chemical shift difference (in Hz) between A and X spins and *n* is the number of X spins. *S*_*A*1_ , *S*_*A*2_ and *S*_*A*3_ (Equation (1) (i) ‐ (iii)) represent the signal contribution arising from A spins, while *S*_*X*1_ , *S*_*X*2_ and *S*_*X*3_ (Equation (2) (i) ‐ (iii)) arise from X spins.

For the plane in which both spins experience the first 90° pulse, nine compartments (3x3) can be identified (compartments 10 to 18, as depicted in Figure 2b (ii)). The first 9 compartments represent the plane in which only A spins experience the first pulse (Figure 2a (ii)) and in compartments 19 to 27 only X spins experience the pulse (Figure 2c (ii)). The signal in these two planes is equivalent to that in the central plane, excluding the contributions related to the spin that is not affected by the first pulse. If the X spins are not affected by the first pulse, the chemical shift dephasing in the last interval is not refocused. With respect to the A spins, their magnetisation would remain oriented along the longitudinal axis after the first pulse and it would not therefore be possible to transfer any magnetisation to the X spins.

Expressions for the signal from each compartment are given in Figure 2 (see Equations (1) and (2)). Their relative contribution to the overall signal depends on the volume of the individual compartments. The ratio between the chemical shift difference and the pulse bandwidth, *k*, determines the volume of each compartment. The expressions for the partial volumes contain the products of *k* or 1 − *k* factors, in which *k* applies when only one of the spins experiences a given pulse and 1 − *k* when both do. The weightings are shown in Figures 2a‐c (ii) for each compartment. In the central slice, at least one factor of (1 − *k* ) is present as all spins are affected by the first pulse. The remaining factors for each compartment are selected according to the spins excited by the second and third pulses. For example, in compartment 16 only A spins are affected by those pulses, which results in an additional *k* ^2^ contribution.

Provided the metabolite of interest is uniformly distributed throughout the excited region (homogeneous case), the net signal *S* is given by:

(3) $$S=S_{X1}-S_{X2}\left(1-k^{2}\right)+S_{X3}k^{2}+S_{A1}\left(1+k^{2}\right)-2S_{A2}k+S_{A3}k^{2}.$$

Note that the net signal is the same as would be obtained from a model that just considers the central plane (two‐dimensional model), and thus the resulting modulation pattern is also equivalent. However, this simplified approach does not fully reflect the spatial origin of the signal contributions.

Note also that when TE is very short (TE*~* 0) or is set to a multiple of 2/J, e.g., TE = 288ms, for the methyl group of lactate, all terms vanish due to the *sin^2^(πJTE/2)* term, except one and Equation (3) simplifies to $S=S_{X1}$. This indicates that the signal is independent of the CSDA effects (not dependent on *k*). For uncoupled spins ( J=0), Equation (3) reduces to S=1/2, reflecting the well‐known fact that the STEAM signal is half the original magnetisation. The analytic expressions of Equations (1) and (2) were implemented in MATLAB (Mathworks, Natick, Massachusetts, USA), with *n* = 3, making it possible to express the amplitude of the lactate doublet as a function of sequence parameters, TE and TM, pulse bandwidth and field strength. The effects of *T*_2_ relaxation were not taken into account in this model. Lactate chemical shifts and J‐coupling constant were taken from.^20^

### 2.2 MR measurements

MR measurements were performed on a 7T Philips Achieva MR system (Philips Healthcare, Best, Netherlands) with a 32‐channel receive head coil (Nova Medical, Wilmington, Massachusetts, USA) and a volume transmit coil (Nova Medical) with a maximum transmit *B*_1_ field of 20 *μ* T. *In vitro* acquisitions were performed to validate the simulations using a spherical phantom (85 mm diameter) that contained metabolites with similar concentrations to those found in normal brain: 10 mM of NAA, 8 mM of creatine, 3 mM of choline, 1 mM of lactate, 6 mM of myo‐inositol, 10 mM of glutamate, 5 mM of glutamine and 2 mM of 4,4‐dimethyl‐4‐silapentane‐1‐sulfonic acid (DSS) as a reference. To illustrate a whole cycle of TM modulation, ^1^ *H* MRS data were acquired from a 30*x*30*x*30*mm* ^3^ voxel positioned in the centre of this phantom using a STEAM sequence (TR/TE = 2400/144 ms, number of averages = 128, spectral bandwidth = 4000 Hz, number of samples = 4096) with a series of TM values (from 16.1 to 17.4 ms and a step size of 0.1 ms). The three 90° pulses (amplitude‐modulated asymmetric and truncated sinc pulses) used in the STEAM sequence had a bandwidth of 3110 Hz (*k* = 0.27). Water suppression was performed with the VAPOR^21^ scheme and unsuppressed water spectra were also acquired before each scan. A second‐order pencil‐beam volume shimming, a Philips routine based on FASTMAP,^22^ was applied across the volume‐of‐interest (VOI) that corresponded to the water frequency and the shifted VOI that corresponded to lactate.

Area integration was used to quantify the lactate doublet (X resonance, 1.26‐1.37 ppm) in the TM series of proton spectra using a home‐built script in MATLAB. All the area values were normalised to the maximum value of the TM series for comparison with the theoretical values.

To demonstrate the applicability of this analytical model of the STEAM sequence *in vivo*, ^1^ *H* MRS data were collected from a voxel (40 x 30 x 25*mm* ^3^) positioned, as depicted in Figure 7, within a healthy human brain (27 years old, female). The subject gave informed consent to the protocol approved by the local ethics committee. Based on the findings from the simulations and *in vitro* experiment, the minimum TM value (16.9 ms), limited by the dephasing gradient duration, that resulted in a maximal inversion of the lactate doublet for a TE = 144 ms, was used. For comparison, a second acquisition with a longer TE, multiple of 2/J (TE = 288 ms) and the same TM was performed. As shown above, the CSDA effects should not contribute to signal cancellation in this case. For these acquisitions, the three 90° pulses had a bandwidth of 2799 Hz. Both spectra were acquired over a period of approximately 11 minutes (number of averages = 264, TR = 2500 ms). The spectra were phase corrected and visualised using MATLAB with 2 Hz of line broadening.

## 3 RESULTS

Expressions for the signal from each compartment are given in Figure 2. Equations (1) and (2) reveal their dependence on TE and TM. The expression derived for the central compartment, where all ^1^ *H* resonances experience the entire pulse sequence, is in agreement with the one published in.^15^

**FIGURE 3:**
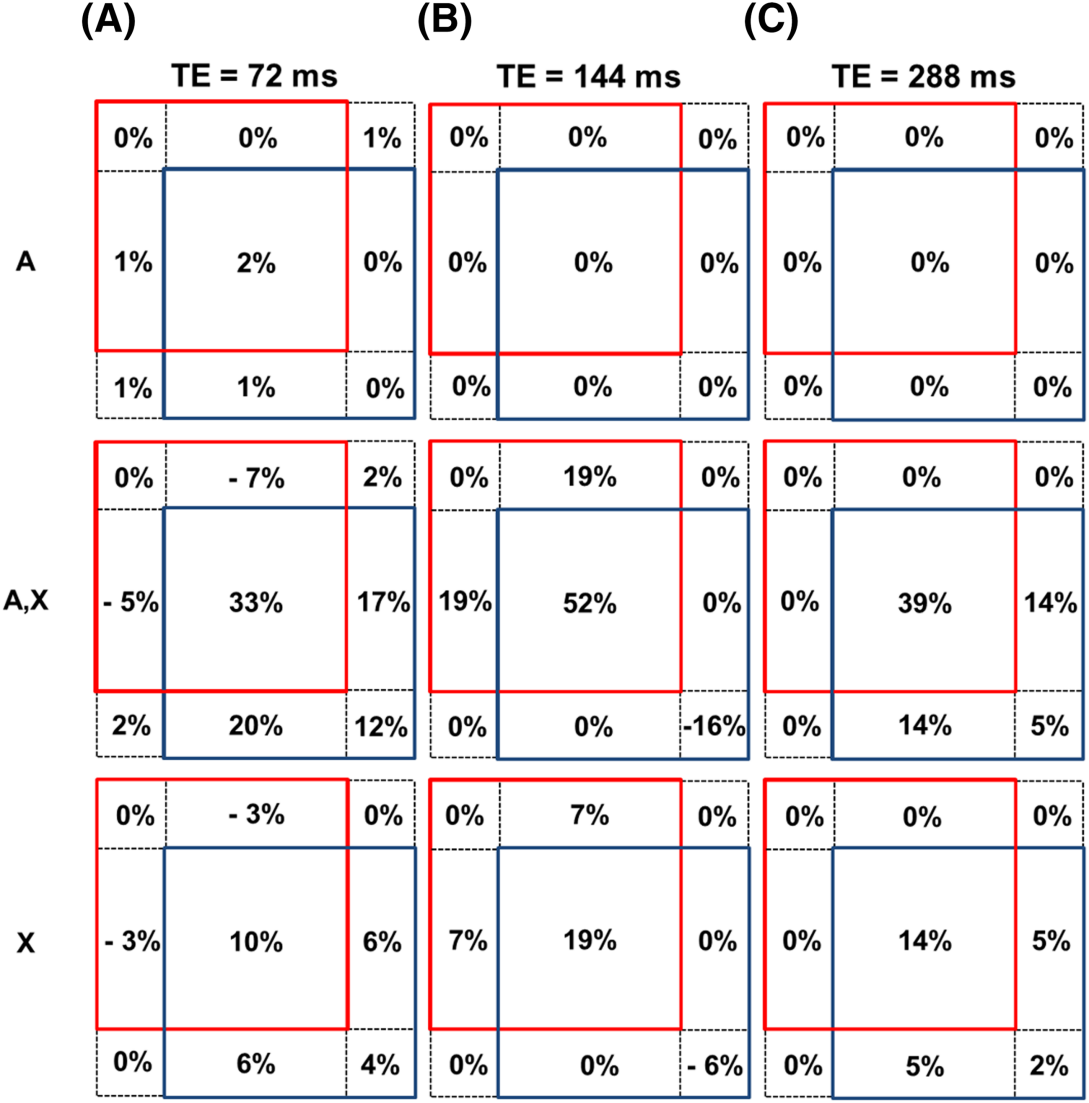
Simulation of the relative contributions for the resulting lactate methyl group signal from each compartment of the three‐dimensional model for TE values of 72, 144 and 288ms in a STEAM sequence. The first row represents the plane where only A spins experience the first pulse, the middle row indicates the central plane, where both chemical groups experience the same pulse and the last row represents the plane where only X spins experience the first pulse. Simulation parameters are TM = 16.9ms, magnetic field strength of 7T and pulse bandwidth of 3110 Hz

Using spectral parameters appropriate for the lactate methyl group at 7T, and a pulse bandwidth of 3110Hz (corresponding to a *k* factor of 0.27), as used in our experimental work, Figures 3a‐c show the location of the compartments and their contributions to the total signal for TE values of 72, 144 and 288ms, respectively.

For a TE value of 288ms, $sin^{2}\left(\pi JTE/2\right)=0$, so all signal components in Equations (1) and (2) vanish apart from *S*_*X*1_ and there is no dependency on TM. For a TE of 144ms, $cos\left(\pi JTE/2\right)=0$, and so *S*_*A*1_ , *S*_*A*2_ , *S*_*A*3_ and *S*_*X*1_ terms all vanish, leaving just *S*_*X*2_ and *S*_*X*3_ terms non‐zero. The total signal, as given by Equation (3), is $S=-S_{X2}(1-k^{2})+S_{X3}k^{2}$, and only the *S*_*X*2_ term has a dependence on TM. For TE = 72ms, most compartments contribute to the total signal.

**FIGURE 4:**
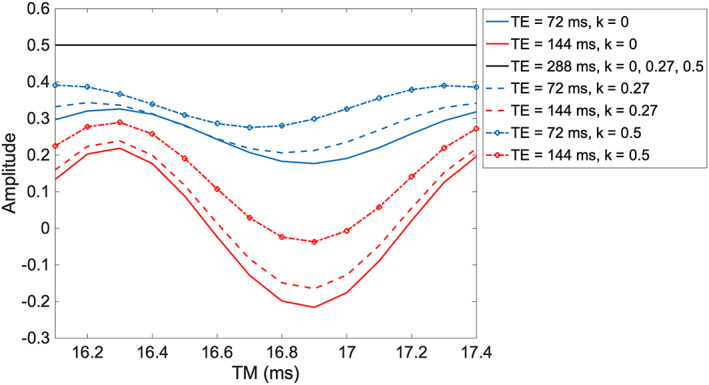
Simulation of the lactate methyl group net signal in the excited volume as a function of TM for TE values of 72, 144 and 288ms, and different values of *k* (*k* = 0.27 corresponds to a pulse bandwidth of 3110Hz) in a STEAM sequence at 7T

The total signal (Equation (3)) is plotted in Figure 4 as a function of TM for the same three TE values and three *k* values: *k* = 0 (no CSDA), *k* = 0.27 (as used in our experimental work) and *k* = 0.5. Note again the lack of dependence on TM and the CSDA for TE = 288ms, and that this yields the maximum signal (in the absence of relaxation effects). When TE = 144ms, an inversion of the lactate peak is obtained, reaching its maximum at TM = 16.9ms. The efficiency of the inversion, i.e., the maximum obtainable negative peak amplitude, is reduced when the CSDA is taken into account (increasing *k* values). For TE = 72ms, the signal is never maximised nor fully inverted, thus diminishing the suitability for *in vivo*applications. Additionally, when TE is set to values in between multiples of 1/J, as in the case of TE = 72ms, the choice of *k* not only affects the amplitude of the TM‐modulated signal, but also the position of its maxima/minima.

**FIGURE 5:**
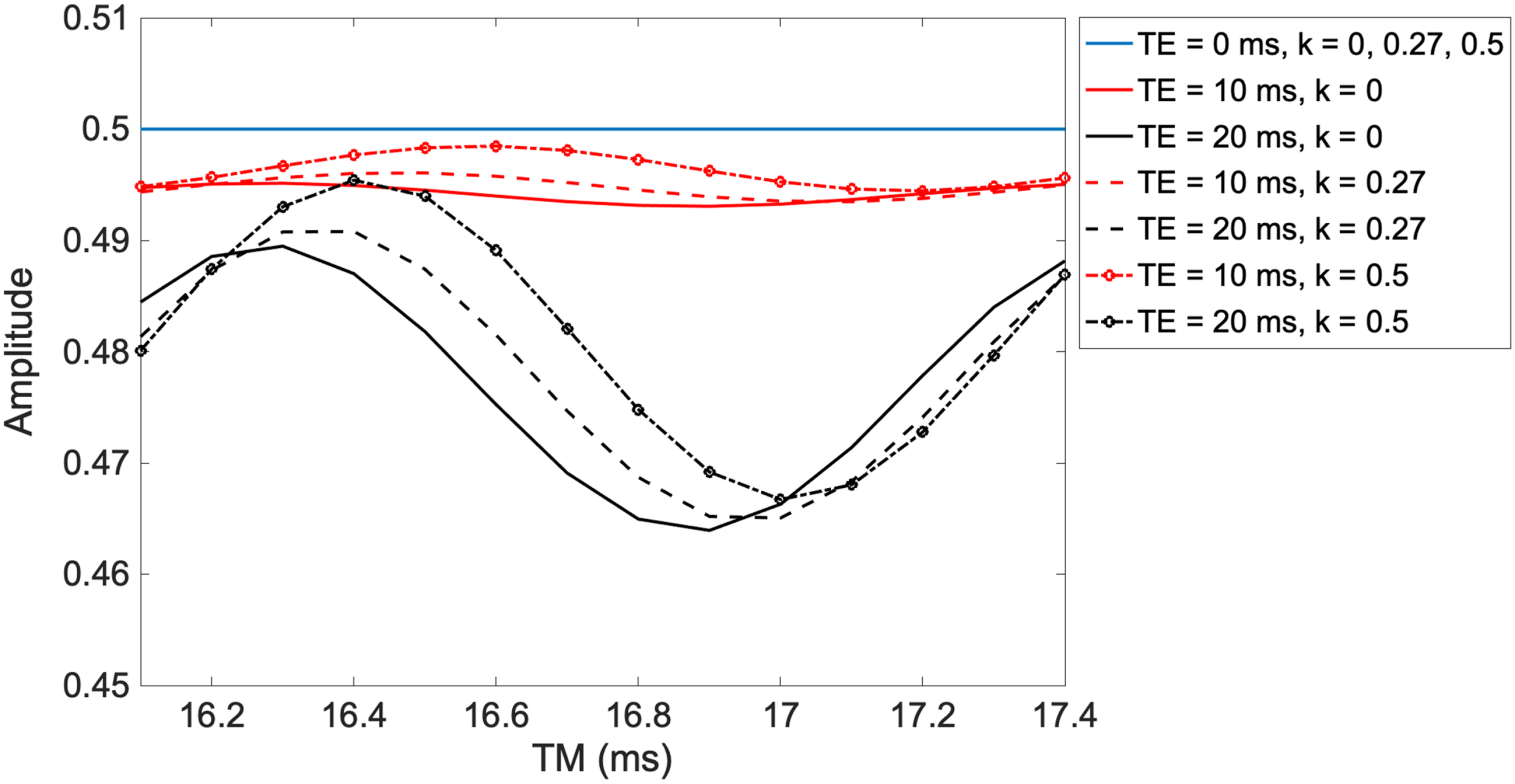
Simulation of the lactate methyl group net signal in the excited volume as a function of TM for TE values of 0, 10 and 20ms, and different values of *k* (*k* = 0.27 corresponds to a pulse bandwidth of 3110Hz) in a STEAM sequence at 7 T

Figure 5 shows the variation of the amplitude of the lactate doublet with TM and *k* for three TE values (0, 10 and 20ms) that represent the typical range of short TE values used at higher field strengths. For TE = 0ms, the maximum amplitude (0.5) is observed irrespective of TM or *k.* For TE = 10ms, variation with TM is observed that is dependent on the amount of CSDA, but the reduction in signal is never more than 1.1%. At TE = 20ms, more significant variation is observed, with a potential loss in signal of about 6.4%.

**FIGURE 6:**
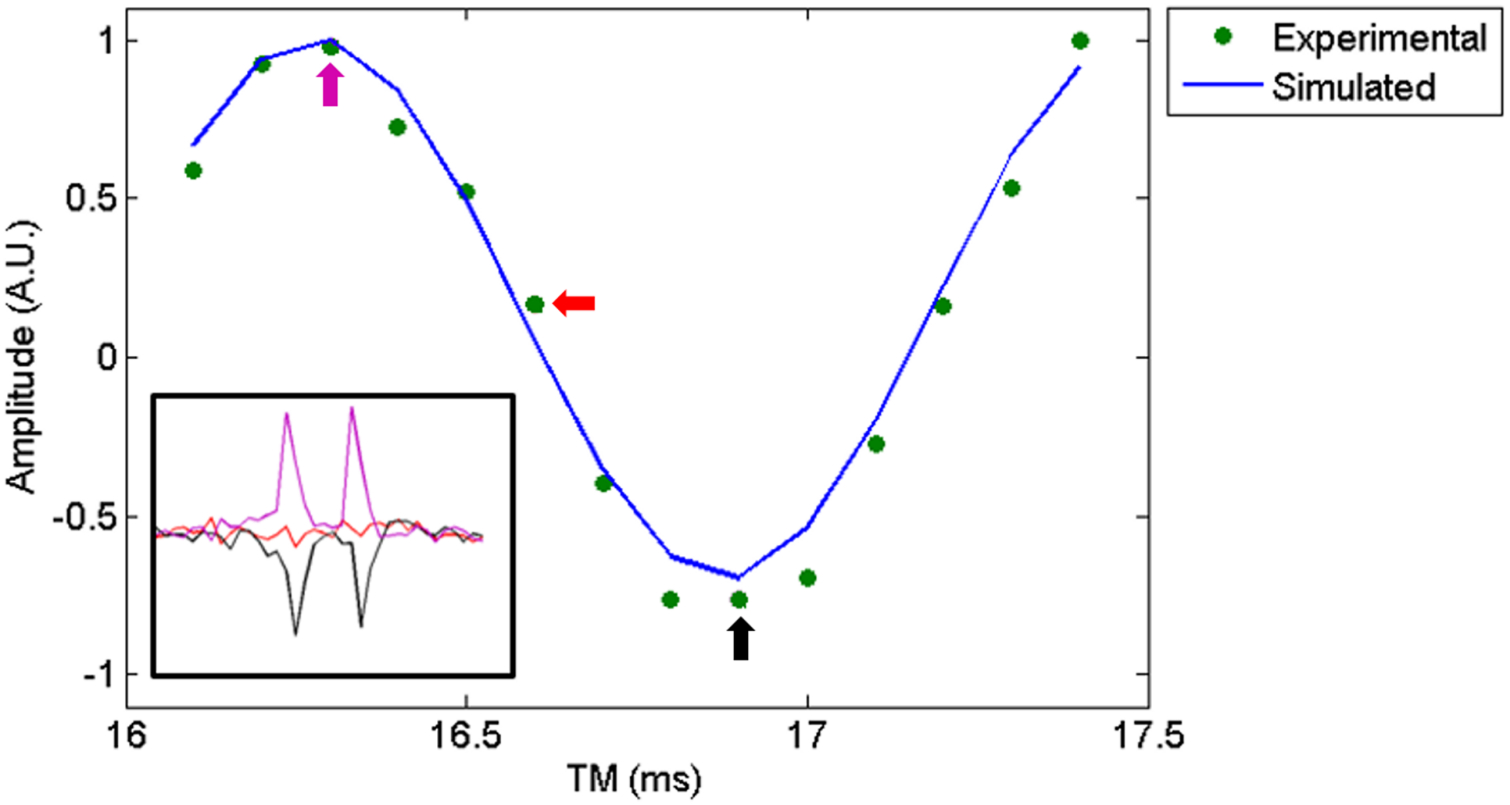
Experimental and predicted TM modulation pattern for the methyl group of lactate. Experimental data were acquired from a phantom with approximate brain metabolite concentrations ([lactate] = 1mM) at 7T using a STEAM sequence (voxel size = 30x30x30 mm^3^ , TR/TE = 2400/144 ms, number of averages = 128 for TM values from 16.1 to 17.4ms). The 90° pulse bandwidth was 3110Hz. Representative spectra zoomed in the lactate doublet are also shown for the key TM values of 16.3 (magenta arrow), 16.6 (red arrow) and 16.9(black arrow) ms

The experimental TM dependence of the total signal for TE = 144ms is shown in Figure 6 (individual points) and compared to the theoretical curve (solid line). Spectra recorded from the phantom at TM values of 16.3 (magenta arrow), 16.6 (red arrow) and 16.9ms (black arrow) are shown in the inset of the figure.

**FIGURE 7:**
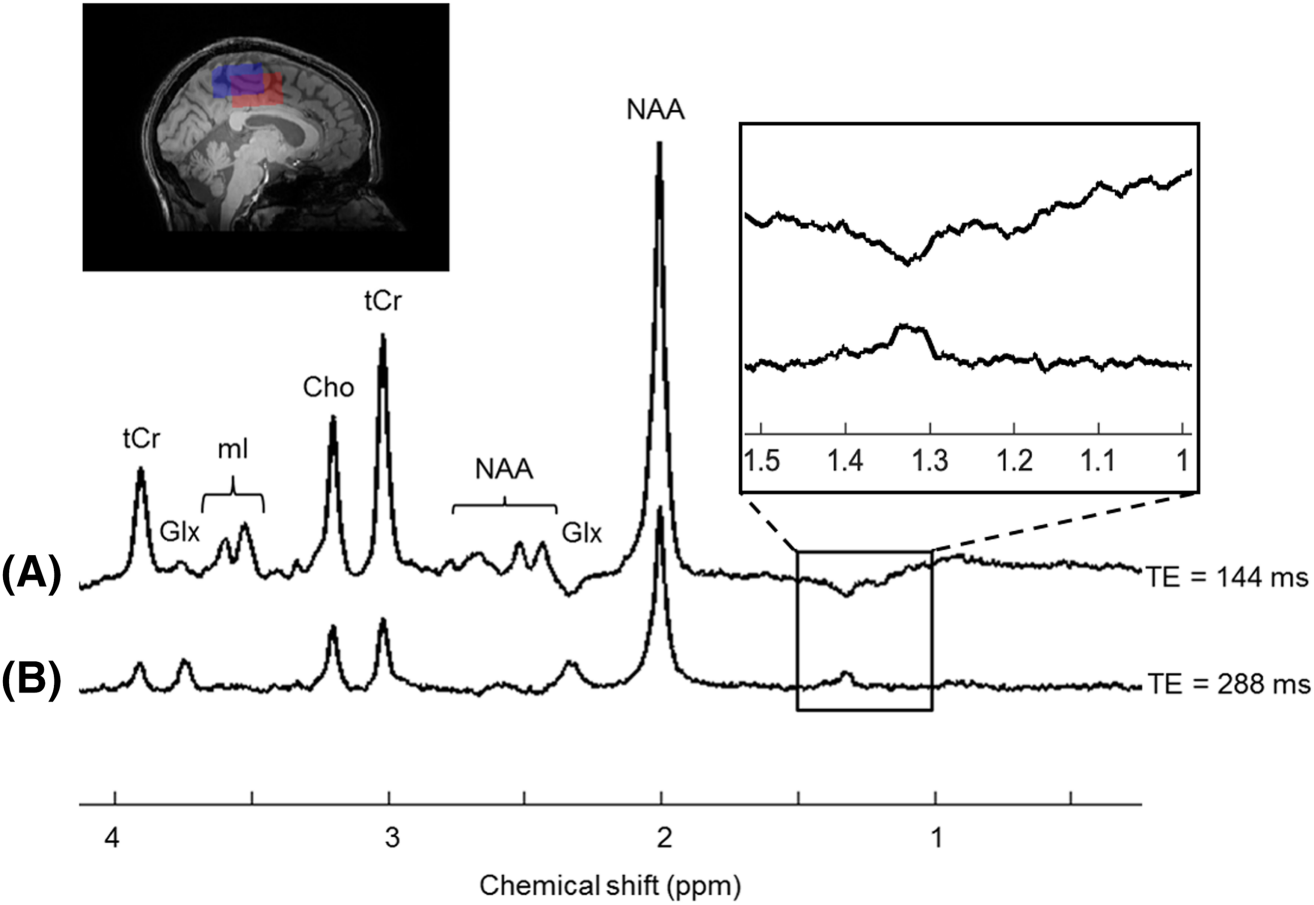
Spectra acquired with the STEAM sequence using a) the optimised TM for the inversion of the lactate (Lac) peak (TR/TE/TM = 2500/144/16.9ms) and b) the optimised TE for attenuation of the CSDA (TR/TE/TM = 2500/288/16.9ms). A 40 x 30 x 25*m* *m* ^3^ voxel was placed in the brain of a healthy volunteer and 264 averages were used to obtain the spectra. The red box represents the voxel at the lactate frequency and blue box the voxel at the water frequency (pulse bandwidth = 2799 Hz). The spectra were phase corrected and displayed in MATLAB with 2Hz of line broadening. Glx represents the combined peaks of glutamate and glutamine and mI refers to myo‐inositol

Figure 7 shows the ^1^ *H* brain spectra obtained from the human volunteer using a TE of 144 ms (upper trace) and 288ms (lower trace) and a TM of 16.9 ms, chosen to give the maximum inverted signal for the lactate doublet at TE = 144 ms (see Figure 6). The measured linewidths of the NAA peak at 2.01 ppm analysed from the spectra without line broadening for the acquisition with TE = 144ms and TE = 288 ms were 11.54 Hz and 11.58 Hz, respectively. The SNR of the same peak was 105 (TE = 144 ms) and 42 (TE = 288 ms). Clear signals from the methyl group of lactate are seen in both cases (inverted in the case of TE = 144 ms).

## 4 DISCUSSION

The quantum mechanical formulation of the coherence pathways in PRESS is relatively straightforward, as the two selective 180° pulses can be treated independently of each other, in contrast with the selective 90° pulses in the STEAM sequence which act in combination to induce a stimulated echo.^18^ In this work, simulations based on the derived analytical expressions in the product operator formalism allowed a better understanding of the spatial distribution of a weakly‐coupled spin system signal, as a function of the STEAM sequence parameters. The simulations reveal that for a homogeneous volume, two‐ and three‐dimensional analyses predict the same total signal and modulation patterns. However, the three‐dimensional analysis indicates where within the excited volume the signal originates and this may be important in the case of an inhomogeneously distributed metabolite.

Considering the case of lactate ( *AX*_3_ ), it was found that for a TE value of 72ms, the methyl group of lactate signal is not confined to a specific plane or set of compartments, while with a TE = 144ms, there is no signal contribution from the plane where only A spins experience the first pulse; however the signal is still distributed throughout the remaining volume. On the other hand, with TE = 288ms, the net signal is restricted to the VOI of the methyl group of lactate, which demonstrates the signal independence of *k* and therefore of the CSDA. The fact that, with this TE value, the signal arises from a region with defined boundaries can be exploited to optimise the positioning of the spectroscopy voxel; if the focus of the measurement is the methyl group of lactate, the VOI for this chemical group should coincide with the region of interest one desires to acquire signal from.

The information on the lactate signal distribution within the excited volume was also used in the work of Edden et al.,^23^ which suggested the combined use of inner volume saturation bands and the PRESS sequence with a TE = 144 ms at 3 T, to remove the contribution of compartments that lead to signal cancellation. In this work, it was shown that with the STEAM sequence some compartments also contribute negatively to the net signal in the cases of TE = 72 and 144 ms. However, a similar strategy as used by Edden et al. cannot be easily applied with the STEAM sequence, as the saturation bands would have to target specific compartments rather than the entirety of the excited region excluding the central compartment as in the PRESS sequence. Moreover, the addition of saturation slabs to only retain the signal from the central compartment, which corresponds to the ‘ideal situation’, would result in a further signal reduction of approximately 50%. Nonetheless, it is possible to observe that at TE = 288 ms, all compartments contribute positively to the overall signal, resulting in a maximal signal (neglecting relaxation effects).

The simulations carried out with the proposed model also allowed the investigation of the signal modulation as a function of TM. It was possible to verify that with TE = 288ms, there is no TM modulation, while for a TE = 144ms, the TM modulation can be used to obtain an inverted lactate doublet. The TM that led to maximal inversion of the lactate peak for this TE, which matched in both theoretical and experimental data, was 16.9ms. However, for a non‐negligible pulse length, it is not possible to achieve a complete inversion, and the inversion efficiency depends on the RF bandwidth.

The performance of the STEAM sequence at TE = 144ms with the optimal TM and at TE = 288 ms was assessed with an *in vivo* acquisition. Despite not being able to achieve 100% inversion efficiency (or equivalently ‐0.25 of the total magnetisation), due to the limited bandwidth and the RF field inhomogeneities at 7T, an inverted lactate peak was easily detectable in a healthy human brain (TE = 144ms). By visual inspection of the spectrum acquired with TE = 288ms, a positive lactate peak could be detected, comparable in magnitude with the one obtained with TE = 144ms.

Additionally, in the acquisition with TE = 288ms, the lactate signal was less affected by lipid and MM contamination, making this measurement less dependent on the individual lipid/MM content. Although it has been suggested that a general MM profile is sufficient for an accurate metabolite quantification at 7T,^24^ individual variability in any of these superimposed signals could contribute to an over‐ or under‐estimation of the lactate concentration.

As first proposed by Lange et al.^25^ for 3T acquisitions with the PRESS sequence, the use of TE = 288ms can be a strategy to overcome the CSDA. Here, the non‐dependence of the signal on the CSDA at TE = 288ms allowed the detection of a clear lactate peak despite the loss due to transverse relaxation decay. Therefore, in acquisitions with the STEAM sequence at 7T, it is recommended to use TE = 288ms, with as short a TM as possible to minimise longitudinal relaxation. However, at higher field strengths than 7T, the use of TE = 144ms might be more advantageous for lactate determination, due to the shorter transverse relaxation times.

Short TE experiments are commonly employed when multiple metabolites are to be measured, and lactate is often determined in this way, for example in functional MRS studies of the response to a visual stimulus.^3^, ^4^, ^5^ Of course, such measurements are challenging in the presence of significant lipid and other macromolecular resonances in the vicinity of the 1.31ppm lactate methyl doublet. We showed that for a TE value of 10ms, there is negligible loss of lactate signal with TM and *k*, and even for a TE of 20ms, the loss is less than 10%. However, as TE increases, the signal variation with TM increases, and this in turn depends on the extent of the CSDA. In such circumstances, if the SNR of lactate is critical, optimisation of TM may be worthwhile. A similar argument applies to other coupled resonances, and their signals could be optimised using the theoretical expressions developed here (for example alanine, also of the *AX*_3_ form).

The effect of the CSDA on J‐coupled spins at ultra high‐field has raised the motivation for studies to provide a quantitative description of the signal loss using a quantum mechanical approach, while proposing several methods to mitigate its influence. This work described the implementation of an analytical model that takes into account the CSDA in the three applied directions of field gradients for a correct determination of the weakly‐coupled spin system signal. Although the use of numerical models brings several benefits, including integration of RF pulse shapes into signal simulations, the advantage of an analytical model is that it provides the full characterisation of the signal origin throughout the excited region and a deeper understanding of the effect of sequence parameters on each coherence pathway. Kaiser et al.^16^ have shown that for weakly‐coupled spin systems, such as lactate, the differences between ideal (non‐localised) simulations and full three‐dimensional simulations with experimental RF pulses are negligible for the STEAM sequence (TE = 70ms at 4T). Further investigation is required to verify whether incorporating pulse shapes into these localised models is crucial for an accurate determination of the lactate signal acquired with longer TEs at 7T.

In conclusion, this work demonstrated the feasibility of detecting lactate at 7T with sufficient SNR and minimal contamination from lipids/MM by using a standard STEAM sequence with optimised timing parameters.

## FINANCIAL DISCLOSURE

This work was supported by the Medical Research Council (MR/K020803). CCF received financial support from the Initial Training Network, HiMR, funded by the FP7 Marie Curie Actions of the European Commission (FP7‐PEOPLE‐2012‐ITN‐316716). CGS received financial support from Fundação de Amparo à Pesquisa do Estado de São Paulo (2013/19340‐6).
